# Climate extremes can drive biological assemblages to early successional stages compared to several mild disturbances

**DOI:** 10.1038/srep30607

**Published:** 2016-08-16

**Authors:** Carlos Sanz-Lázaro

**Affiliations:** 1 Department of Biology, University of Pisa, CoNISMa, Via Derna 1, Pisa, Italy

## Abstract

Extreme climatic events have a major role in the structuring of biological communities, and their occurrence is expected to increase due to climate change. Here I use a manipulative approach to test the effects of extreme storm events on rocky mid-shore assemblages. This study shows that an extreme storm can cause more negative effects than several mild storms, primarily by bringing the biological assemblages towards early stages of succession. This finding contrasts with the effects of clustering of climatic events due to climate change, which are expected to mitigate its ecological impacts. Thus, the ecological consequences of climatic events that are influenced by climate change may have contrasting effects depending on the features that are considered. These results have relevant implications in the forecasting of the ecological consequences of climate change and should be considered when designing measures to mitigate its effects.

Predictions of the effects of climate change are a major focus of research, as they can help to assess its likely effects on ecosystems, and thus to develop appropriate preventive and mitigation measures^1^. Most research in this field has focused on changes in the trends of mean climate values, rather than the occurrence of extreme events^2^. Extreme events are rare climatic events with an abnormally high intensity^3^,^4^, and climate change is expected to increase their occurrence^5^,^6^,^7^. Extreme events can have a disproportionately-high impact on ecosystems relative to the short time scale at which they occur^8^,^9^. Thus, extreme events are expected to be important drivers structuring biological assemblages^10^,^11^, and consequently on ecosystem functioning.

Understanding the structure and variability of biological assemblages is a primary challenge in ecology^12^. The drivers of ecological change, such as extreme events, are to some extent known^13^,^14^, but more research is needed to understand how changes in the characteristics of these drivers can affect the structure of biological assemblages. Manipulative experiments are an effective tool to simulate the effects of current and future predicted extreme events, and to study their consequences on biological assemblages. They allow us to identify cause-effect relationships, and to include appropriate controls that are not possible in field observations^2^.

Frequency and intensity are major factors that determine the effects of extreme events^11^. Studies dealing with the effects of disturbances on biological assemblages have manipulated frequency, both alone^15^ and in combination with intensity in a fully factorial design^16^, keeping a constant intensity per disturbance event for each level of intensity but a variable overall intensity. Experiments related to climate change have focused on the temporal patterns of disturbances, thus keeping the same overall intensity of disturbances constant. Some modelling studies have tested the effects of the clustering of hurricanes^17^. Other studies have been manipulative and have simulated disturbances under factorial designs by modifying the temporal patterns of sediment loading and eutrophication in streams; and the temporal patterns together with the intensity of storms in rocky shores^18^,^19^ and of rainfall in grasslands^20^.

In contrast, the effects of extreme events on biological assemblages have received much less attention, despite their importance in structuring biological assemblages and the influence that climate change can have on them. New methodologies are needed to improve our understanding on extreme events, complementing currently used factorial designs based on the inherent parameters of extreme events. By using a combination of inverse levels of intensities and frequencies, but keeping the same overall intensity, we could simulate a gradient of increasing severity in climatic events which would allow us to examine the trend and possible thresholds of the responses of the biological assemblages (*sensu* Smith)^21^. In addition, experiments should be repeated at different times of the year, because organisms can have different responses depending on the time of the year at which the extreme event occurs^22^. The combination of these approaches would help to increase our understanding of the ecological consequences of extreme events.

Marine habitats are threatened by a wide range of anthropogenic activities. In rocky shores, storms play a key role in structuring biological communities because they generate empty patches^23^, thus favouring colonization. Consequently, these habitats will be sensitive to increases in the severity of mechanical disturbances caused by increasingly extreme storms due to climate change^24^. Rocky shores have a number of characteristics that facilitate ecology studies^25^, such as accessibility, ease of developing controlled experiments, a simple spatial layout (they are essentially a two dimensional habitat), and the high population turnover of many species. Thus, rocky shores are an ideal system to help us study the effects of extreme events on biological assemblages.

The aim of this study is to assess the effects of climatic extremes on biological assemblages, measuring the effects of storms on rocky shores as a test case. I started by developing a factorial design to test the effects of frequency and intensity and their possible interactions. Based on this, I produced a gradient of scenarios in which storm events became more extreme (i.e. increasing the intensity and decreasing the frequency), by inversely varying frequency and intensity but maintaining the overall intensity of storms as a constant. The experiment was run twice, beginning at different times of the year to take into account of temporal variability. My hypothesis was that late-colonising species would be more affected by an extreme event than early-colonising species, because late colonisers have a longer recovery time than early colonisers. Consequently, under extreme events the cover of late colonisers will be reduced, allowing the proliferation of early colonisers. This will result in assemblages at earlier stages of succession compared to assemblages suffering more frequent but less extreme events.

## Results

**Figure 1:**
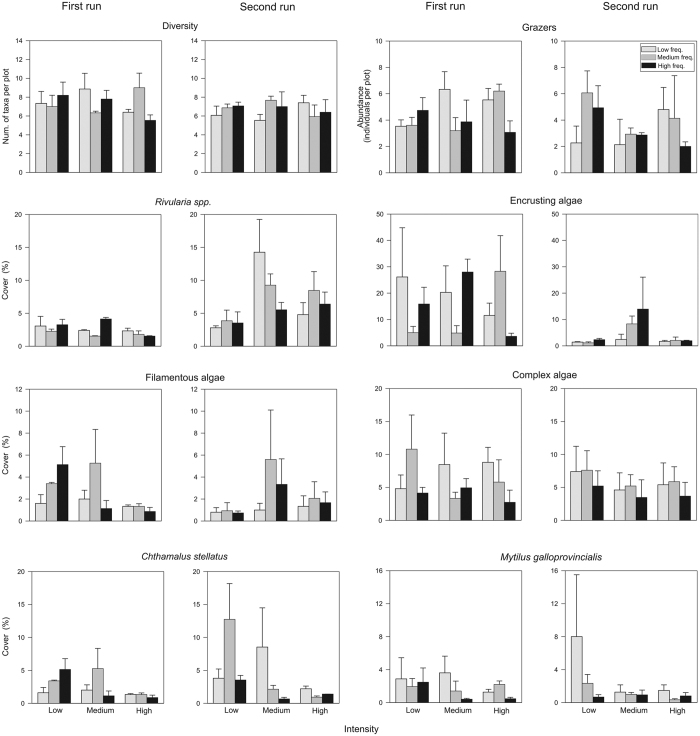
Changes in diversity, grazer abundance and cover of groups and individual taxa of mid-shore rocky assemblages according to different levels of intensity and frequency of simulated storm events (mean ± SE; n = 3). The values of each replicate correspond to the mean of the five samples from each experimental plot. The experiment was run twice, each beginning at a different time of the year.

A two factor ANOVA comparing the *intensity* and *frequency* of events indicated that *Chthamalus stellatus* cover had significant differences for *intensity*. This was also the case for *Rivularia spp.,* but only for a certain period of the year. *C. stellatus* cover was significantly reduced under events of high intensity compared to low intensity events, while the growth of *Rivularia spp.* was favoured at intermediate intensity (Appendix 1). The range of the values of the response variables was not notably influenced by the time of year, except for *Rivularia spp.* and encrusting algae (Fig. 1). There were no significant differences for *frequency* or the interactions between factors for any of the biological groups studied.

**Figure 2:**
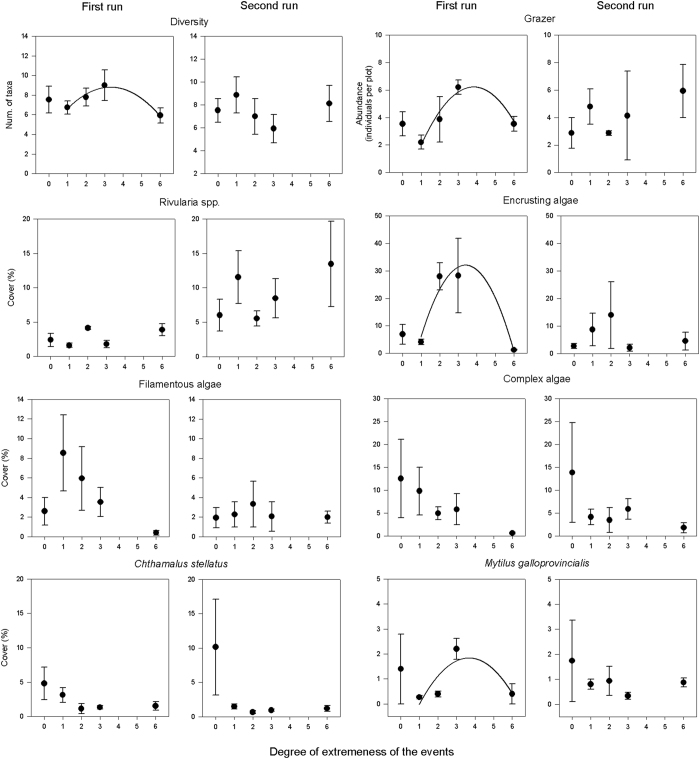
Changes in diversity, grazer abundance and cover of different taxa of mid-shore rocky assemblages along a gradient of simulated storms with the same overall intensity, ranging from several small storms to a single large one (i.e. storms become more extreme) (mean ± SE; n = 3). The values of each replicate correspond to the mean of the five samples from each experimental plot. Level 0 corresponds to the unmanipulated experimental plots. Curves show significant regressions along the gradient excluding level 0 (Table 2). For a schematic representation of the experimental design see Appendix 4. The experiment was run twice, each beginning at a different time of the year.

In undisturbed plots, most biological groups responded similarly and were not influenced by the time of year. Only encrusting algae, *Rivularia spp.* and *C. stellatus* had differences in average cover depending on the time of the year, with values that were 2.6, 2.5 and 2.1 times higher, respectively (Fig. 2).

When comparing control plots along the gradient of increasing storm intensity, significant differences were found for *C. stellatus*. Similarly, the cover of complex algae was notably higher in the undisturbed plots than in the treated plots. The cover of filamentous algae and *Rivularia spp.* was sometimes greater in the disturbed than in the undisturbed plots. Encrusting algae cover tended to be high in disturbed plots, while the opposite was found for *Mytilus galloprovincialis* (Table 1; Fig. 2).

Many response variables (diversity, grazer abundance; encrusting algae and *M. galloprovincialis* cover) had significant second-order polynomial relationships along the gradient of increasing storm intensity, for certain time of the year, peaking at the midpoint of the gradient. At the same time of the year, filamentous and complex algae showed a negative trend as storms became more intense and less frequent (Table 2). In general, the values of the response variables had similar ranges, independent of the time of the year. In the most extreme event, the cover of sessile taxa was low compared to undisturbed conditions, with *Rivularia spp*. the only exception (Fig. 2).

**Figure 3:**
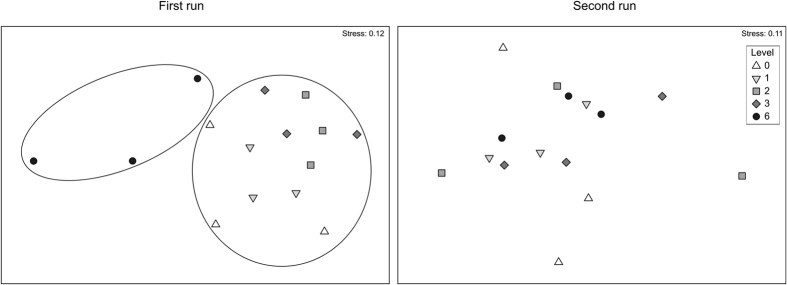
Non-metric multidimensional scaling (NMDS) based on the average values of cover over time of sessile taxa for each sampling plot. The values of each replicate correspond to the mean of the five samples from each experimental plot. Treatments comprise a gradient of simulated storms with the same overall intensity, ranging from several small storms (level 1) to a single large one (level 6). Level 0 corresponds to the unmanipulated plots. For a schematic representation of the experimental design see Appendix 4. The experiment was run twice, each beginning at a different time of the year. Circles separate samples that are significantly different according to PERMANOVA (appendix 2) and post-hoc comparisons (appendix 3).

The structure of the community of the control plots was not significantly different from the treated plots that embraced the gradient of increasing storm intensity, although in some cases differences were notable (Appendix 2). The plots that have suffered the most-extreme storm events were significantly different from those the plots with milder storms and undisturbed plots for a certain period of the year (Fig. 3; Appendix 3).

## Discussion

This study demonstrates that the *intensity* of storms has a greater effect on the abundance of certain taxa on the rocky shore in comparison to *frequency*, particularly for the late-colonising species *C. stellatus*, and the early-colonising taxon *Rivularia spp.*; this corresponds with the results of similar studies^26^. Late colonisers (*C. stellatus* and complex algae) were usually negatively affected by storms, particularly extreme events. Conversely, early colonisers (filamentous algae, *Rivularia spp*. and encrusting algae) were neutrally or positively affected by storms. This indicates that the disturbance produced by storms can increase the abundance of early colonisers, possibly by diminishing the cover of late colonisers, providing an opportunity for establishment^27^. In contrast, the diversity of sessile taxa had positive and negative variations compared to undisturbed conditions, indicating no relationship between stability and diversity in this habitat, as also found by Cusson *et al*.^28^.

The strength of the biological trends over the gradient of storm intensity was influenced by the time of the year. In some cases, the ecological structure of the assemblage that suffered the most intense storm was significantly different to the structure of the other treatments, but in others there was no significant difference in ecological structure between treatments, even though they differed visibly from the ecological structure of the control. For specific biological groups, there was a succession in the predominance of early colonisers along the gradient of increasing storm intensity, which varied slightly according to the time of the year that the experiment was performed.

This temporal variation had different influences on the effects of extreme events, which could be because many species have seasonally-constrained periods of reproduction, recruitment and growth during the year^29^. An increase in habitat availability and the reduction of direct competition due to climatic events such as storms, can favour the proliferation of certain species if they occur during their reproductive or recruiting periods^30^. Additional runs of the experiment over multiple years would have given a clearer picture of inter-annual variability. However, it is expected that there is a wider variation within the year than among years, due to the known seasonality of these algae assemblages. Nonetheless, some taxa showed patterns that were not time dependent.

In comparison to the treatment with several mild events, the cover of late colonisers (*C. stellatus* and complex algae) was highest in the control, and lowest in the treatment with the most extreme event. Specifically, the cover of complex algae reached the lowest values in the plots that suffered the most extreme events, in comparison to other plots that suffered more climatic events but with less intensity. The cover of sessile taxa was much lower for the scenario of one extreme event in comparison to the scenario with several mild climatic events, for which the only exception was the early colonisers, (encrusting and filamentous algae and *Rivularia spp.*) which remained stable or increased. These taxa are characterized by very quick recovery rates, particularly for *Rivularia spp.* which are cyanobacteria. Therefore, this study demonstrates that an extreme event can have more negative impacts than several mild events, directing the biological assemblages towards earlier stages of succession.

Recently, there has been a notable increase in our understanding of the effects of climate change, in terms of the expected modifications of the temporal patterns of climatic events. The clustering of climatic events produced by climate change is expected to ameliorate the deleterious effects of these events on biological assemblages^17^,^18^,^26^,^31^. Scenarios with clustered climatic events are expected to keep the ecosystem in a later successional stage (i.e. with a higher proportion of late colonising species), for a longer time span than scenarios with climatic events of constant frequency^17^. However, the results of our study are not consistent with this hypothesis. The reason for these differences may be that the aim of this study was different, focusing on the effect of climatic events becoming more extreme and not on the clustering of climatic events with the same intensity.

In this experiment, the most intense perturbation left completely bare rock, which can happen under heavy storm conditions^26^. The bare rock cannot be directly colonized by most macroscopic intertidal organisms without the prior development of a biofilm. Biofilms are composed of bacteria, algae and fungi which produce an extracellular polymeric substance matrix^32^ that favours the attachment of propagules of sessile macroorganisms^33^. Thus, this is a form of primary succession in terrestrial ecology in which no organic matter, live plants or propagules remain after the disturbance. In this case, primary succession takes longer than secondary succession^34^. This could explain why the effects of an extreme event are different to the effects of several climatic events of a mild intensity. Consequently, extreme events of sufficient magnitude are likely to force the ecosystem towards primary succession, resulting in long recovery times^35^.

The design of this experiment comprises an orthogonal design based on the two main attributes that define extreme events - intensity and frequency - and a gradient of climatic events ranging from several small events to a single large one with the same overall intensity. It has been argued that orthogonal designs of intensity and frequency may not be appropriate to test the effects of climate change (at least when it is related to temporal variation of climatic events) because the overall intensity can differ between treatments^36^. However, when the focus of the study is extreme events, which are defined by intensity and frequency, this type of analysis is the only way to test for possible interactions among them. Nonetheless, this experimental design has different overall intensity among treatments and complementary tests should be performed, so the second part of the experimental design of this study has allowed us to account for this issue. This part of the experimental design is a robust tool to study the effects of extreme events on biological assemblages for a number of reasons. Firstly, the use of a gradient allows us assess the trends and thresholds of disturbances produced by climatic events as they become more extreme. Secondly, the use of same overall intensity for each treatment allow us to ensure that the results obtained are not confounded by variation in the total intensity between treatments. Thirdly, the mixed-effects model technique used in the analyses allows us to model the temporally-autocorrelated observations within experimental units and explore univariate succession trends. Finally, multivariate routines allow us to distinguish groups with different ecological structure and explore the successional trends of the whole community.

The two parts that comprise the experimental design performed in this study are complementary and help to develop our understanding of the ecological consequences of extreme events in biological assemblages. The experimental framework described in this study provides a novel tool that complements previously methods^21^ and could be used in other habitats simulating this or other climatic events. The replication of this experiment in other habitats and with other types of extreme events would help us to find out about the generalities of the findings of this study.

This experiment, along with other studies of the patterns of disturbances, demonstrates that global change may have very different consequences depending on the parameter that is considered. All of the effects of climate change should be considered in combination, in order to give the most-accurate prediction of the derived outcomes, and to develop the most suitable preventive and adaptive measures to account for climate change.

In conclusion, the intensity of climatic events (such as storms in rocky shores) is expected to have a greater effect than frequency, mainly by reducing the cover of late colonisers. An extreme event may have more deleterious effects than several mild events, because the former leads the biological assemblages towards early stages of succession, although the successional patterns may be temporally/seasonally dependent. This study helps to broaden our perspectives of the ecological consequences of changes in the occurrence of climatic events derived from climate change, with contrasting effects depending on the feature considered. While the clustering of climatic events is expected to mitigate the ecological impacts of disturbances, the increasing occurrence of extreme events is expected to exacerbate these impacts. These results have implications for predictions of the ecological consequences of climate change, and should be considered when designing measures to mitigate its effects. The experimental design used in this study could be applied to other habitats and with other types of climatic events.

## Methods

### Study area

The experiment took place at Calafuria, a sandstone rocky shore located in the Ligurian Sea, Italy, NW Mediterranean (43° 30′ N, 10° 20′ E), between 0 and 0.5 m above the mean low-water level. This assemblage is representative of exposed mid-shore habitats of rocky shores in the north western Mediterranean. At this height on the shore the red alga *Rissoella verruculosa* (Bertoloni) J. Agardh predominates. The upper limit of the assemblages is dominated by the barnacles *C. stellatus* (Poli) and cyanobacteria (*Rivularia spp.*), while the bottom limit of the assemblage is dominated by macroalgae, mainly *Cystoseira compressa*, filamentous, encrusting, coarsely branched, articulated corallines and foliose algae. The main grazers at this height on the shore are *Patella ulyssiponensis*, *P.rustica and Porchus turbinatus*^37^.

### Experimental design

Experimental plots (50 × 50 cm) were randomly distributed along 3 km of the shoreline, delimited with epoxy putty (Subcoat S; Veneziani, Trieste, Italy). Disturbances were produced by eroding the rock by means of a chisel mounted on a battery drill (Tanaka, Aubum, Washington, USA), which causing the mechanical removal of sessile organisms. The levels of disturbance consisted of one, two, three and six drill passes along the plot, respectively. To help visualize this, one drill pass along the plot left *c.* 75% of the initial cover of sessile organisms, while six drill passes left the rock completely bare of any organisms; this occurs naturally when very heavy storms break rocks, creating new substrata^23^. This is a standardized procedure that mimics the effect of storms generating empty patches on rocky shores^26^. As regards the frequency of the disturbance, it consisted of four levels: one, two, three and six simulated storms during six months. The levels of frequency were chosen based on the records of storm surges over the previous decades in this area (0–6 storms per year)^38^.

The experiment consisted of two parts. Part 1 of the experiment aimed to test the effects of intensity (per disturbance event), frequency of the disturbance and their possible interactions in the biological assemblages. To do this, a fully factorial design was performed comprising the levels of the factors *intensity* and *frequency* that are likely to occur in nature. The factor *intensity* included 3 levels: one, two and three drill passes over the plot. The factor *frequency* included also 3 levels: one, two or three storms during six months (see Appendix 4; part 1 of the experiment). The level 6 for the factors *intensity* and *frequency* could not be included in this design since very intense storms are only likely to occur with more than a very low frequency, while very frequent storms are expected to be of a very low intensity.

Part 2 of the experiment aimed to test the effect of storms becoming more extreme (i.e. increasing the intensity and decreasing the frequency) on the biological assemblages. To do this, a gradient of scenarios of storm events becoming more extreme, which are likely to occur in nature, was created by inversely varying frequency and intensity, while keeping the overall intensity of storms constant. The gradient comprised the treatments from part 1 of the experiment with level two of intensity and three of frequency and, vice versa; and two more treatments, with level six of intensity and level one of frequency, and vice versa (see Appendix 4; part 2 of the experiment). In total the experiment comprised 11 treatments, each having three replicates.

To examine possible generalities of the results, the experiment was run twice, starting at a different time of the year, because the species cover of rocky shore assemblages is highly variable during the year^29^. Thus, for each run, a total of 33 experimental plots was deployed, in addition to three unmanipulated plots that were used as references of undisturbed conditions. The first run started on August 2011 and the second run started on March 2012. In the first run of the experiment, continuous bad weather conditions prevented treatment and sampling in December (Appendix 5 shows the temporal distribution of sampling and simulated climatic events during the experiment).

### Sampling

Each quadrant was sampled five times: the first sampling took place two months after the first disturbance, and then each following month after the last disturbance. Sampling was always carried out before applying a disturbance, so that all plots have not been disturbed for at least one month before sampling (see Appendix 5). To avoid possible border effects the sampling was carried out in the centre of the experimental plot in an area of 20 × 20 cm. The response variables measured were diversity (measured as number of sessile taxa per plot), abundance of grazers and cover of algae, *C. stellatus* and *M. galloprovincialis*.

### Data analyses

For part 1 of the experiment (see Appendix 4) I carried out a fully factorial ANOVA design, taking both intensity and frequency of events as fixed factors having all possible combinations from levels one to three. For this analysis I used the mean of the five samplings done on each plot. Homogeneity of variance was checked using a Cochran test before performing the analysis. If this assumption was violated then a suitable transformation was applied (see Appendix 1 for the specific transformation used in each case) and then the Cochran test was run again. After running the two-way ANOVA, if significant differences were found, the post-hoc test Student–Newman–Keuls (SNK) was performed^39^.

Then, for part 2 of the experiment I created a gradient of storms that went from several small events to a single large one (i.e. the intensity of the storm is inversely proportional to the frequency) with the same overall intensity (six drills passes along the plot) to avoid confounding effects. I used a mixed-effects model to model the temporally-autocorrelated observations within experimental plots and to analyse this gradient, which included the levels: one, two, three and six, related to the increasing levels of intensity of the storm. So, for example, in the treatment level with the highest degree of intensity (six) I simulated only one storm that occurred at a very high intensity, while in the treatment level with the lowest degree of extremeness (one), I simulated a storm every month that occurred at a low intensity (see Appendix 4, part 2 of the experiment). First, for each response variable I calculated the AICc (corrected Akaike Information Criterion) for the linear and quadratic polynomial regression and choose the model with the lowest AICc value. Then the chosen models were fitted with restricted maximum likelihood (REML) and tested whether residuals conformed to parametric assumptions through quantile-quantile plots and plots of residuals *vs.* fitted values^40^. Then, I refitted the models with maximum likelihood (ML) to estimate the fixed parameters. Furthermore, a planned orthogonal contrast was used to test whether there were significant differences in the response variables between undisturbed and disturbed plots from the gradient of simulated storm intensity. To do this I included the control plots and compared them with the rest of the plots that were used in the mixed-effects model.

In addition, I performed non-metric multidimensional scaling (NMDS) as an exploratory approach to view the spatial ordination of the sampling plots according to the average values of the cover of sessile taxa over time. A permutational multivariate analysis of variance (PERMANOVA) was performed to test significant differences among treatments. First, I used the orthogonal contrast mentioned above between undisturbed and disturbed plots. Then, if relevant, I performed a *post-hoc* test to find pairwise significant differences between treatments. When there were not enough samples for PERMANOVA to accurately estimate the p value, it was calculated using the Monte Carlo test^41^. All the multivariate analyses were based on Bray-Curtis untransformed dissimilarities.

Univariate analyses were run in the statistical environment R (v. 2.15.0). The two-way ANOVA was carried out using the statistical package GAD and the mixed-effects models using the statistical package *nlme*^42^. Multivariate analyses were performed with the software package Primer (v6) and its complementary package PERMANOVA + (v. 1). All statistical tests were conducted with a significance level of α = 0.05.

## Additional Information

**How to cite this article**: Sanz-Lázaro, C. Climate extremes can drive biological assemblages to early successional stages compared to several mild disturbances. *Sci. Rep.* **6**, 30607; doi: 10.1038/srep30607 (2016).

## Supplementary Material

Supplementary Information

## Figures and Tables

**Table 1:** Summary of the planned orthogonal contrasts between unmanipulated plots and treated plots along the gradient of simulated storm intensity (see Appendix 4, part 2 of the experiment for a schematic representation of the experimental design) on the diversity of sessile organisms and the abundance of the following species groups of rocky shore assemblages in both runs of the experiment (df_num_ = 1, df_den_ = 13). Significant differences are indicated in bold.

	First run		Second run	
	Coefficient (SE)	*P*	Coefficient (SE)	*P*
Diversity	−0.03 (0.25)	0.90	−0.01 (0.29)	0.97
Grazers	0.08 (0.24)	0.74	0.31 (0.37)	0.41
*Rivularia spp*.	0.09 (0.18)	0.63	0.75 (0.77)	0.35
Encrusting algae	1.70 (1.94)	0.40	0.92 (1.27)	0.48
Filamentous algae	0.40 (0.58)	0.45	0.1 (0.28)	0.73
Complex algae	−1.45 (0.96)	0.16	−2.01 (0.98)	0.06
*Chthamalus stellatus*	−0.6 (0.25)	**0.03**	−1.81 (0.58)	**<0.01**
*Mytilus galloprovincialis*	−0.12 (−0.12)	0.47	−0.2 (0.15)	0.20

**Table 2:** Summary of the trends of diversity of sessile organisms and abundance of the following species groups of rocky shore assemblages over a gradient of simulated storm intensity (see Appendix 4, part 2 of the experiment for a schematic representation of the experimental design).

		First run		Second run	
		Coefficient (SE)	***P***	Coefficient (SE)	***P***
Diversity	Intercept	4.29 (1.82)	**0.02**	11.65 (2.59)	**<0.01**
	Linear component	2.72 (1.28)	0.06	−3.22 (1.82)	0.11
	Quadratic component	−0.41 (0.17)	**0.04**	0.44 (0.25)	0.11
Grazers	Intercept	−1.56 (1.69)	0.36	3.29 (1.63)	0.05
	Linear component	4.06 (1.19)	**<0.01**	0.38 (0.46)	0.43
	Quadratic component	−0.53 (0.16)	**<0.01**	–	–
Bacteria	Intercept	1.94 (0.7)	**<0.01**	7.22 (3.41)	**0.04**
	Linear component	0.3 (0.2)	0.16	0.84 (0.97)	0.40
	Quadratic component	–	–	–	–
Encrusting algae	Intercept	−20.4 (13.05)	0.12	11.11 (5.87)	0.06
	Linear component	30.97 (9.19)	**<0.01**	−1.28 (1.66)	0.46
	Quadratic component	−4.57 (1.24)	**<0.01**	–	–
Filamentous algae	Intercept	9.17 (2.57)	**<0.00**	2.82 (1.25)	**0.03**
	Linear component	−1.50 (0.73)	0.07	−0.13 (0.35)	0.71
Complex algae	Intercept	10.2 (2.59)	**<0.01**	5.17 (1.72)	**<0.00**
	Linear component	−1.62 (0.73)	0.05	−0.45 (0.49)	0.38
*Chthamalus stellatus*	Intercept	4.59 (1.34)	**<0.00**	1.94 (0.58)	**<0.00**
	Linear component	−1.92 (0.95)	0.07	−0.68 (0.41)	0.13
	Quadratic component	0.24 (0.13)	0.10	0.09 (0.06)	0.13
*Mytilus galloprovincialis*	Intercept	−1.67 (0.76)	**0.03**	0.72 (0.29)	**0.02**
	Linear component	1.91 (0.53)	**<0.01**	0 (0.08)	0.96
	Quadratic component	−0.26 (0.78)	**<0.01**	–	–

Significance coefficients of the regressions are indicated in bold. The experiment was run twice, each beginning at a different time of the year.
