# Supplementary material for: Climate extremes can drive biological assemblages to early successional stages compared to several mild disturbances
Source: Sci Rep. 2016 Aug 16;6:30607. doi: 10.1038/srep30607 (PMC4985811; doi:10.1038/srep30607)

Supplementary information of: “Climate extremes can drive biological assemblages to early successional stages compared to several mild disturbances”

Carlos Sanz-Lázaro^1,2^*

^1^ Department of Biology, University of Pisa, CoNISMa, Via Derna 1, Pisa, Italy

^2^ Present address: Departamento de Ciencias del Mar y Biología Aplicada, Universidad de Alicante, PO Box 99, E-03080 Alicante, Spain

* Corresponding author:

E-mail: [carsanz@ua.es](mailto:carsanz@ua.es) (primary e-mail); [carsanzla@gmail.com](mailto:carsanzla@gmail.com) (secondary e-mail)

Appendix 1. Summary of the two-way ANOVA testing the effects of intensity (three levels: low, medium and high) and frequency (three levels: low, medium and high) of the simulated storm events (part 1 of the experiment; see Appendix 4 for a schematic representation of the experimental design) on the diversity of sessile organisms and the abundance of the following species groups of mid-shore rocky assemblages. When significant differences were found the corresponding SNK post-hoc test was applied. Significant effects are indicated in bold. The experiment was run twice, each beginning at a different time of the year. C: Cochran’s test statistic.

|  | First run |  |  |  |  |  | Second run |  |  |  |  |
| --- | --- | --- | --- | --- | --- | --- | --- | --- | --- | --- | --- |
|  | Source of variation | df | MS | F | p |  | Source of variation | df | MS | F | p |
| Diversity | Intensity=int | 2 | 1.2 | 0.3 | 0.74 |  | Intensity=int | 2 | 0.1 | 0.0 | 0.98 |
|  | Frequency=freq | 2 | 0.3 | 0.1 | 0.92 |  | Frequency=freq | 2 | 0.7 | 0.3 | 0.77 |
|  | Int x freq | 4 | 7.7 | 2.0 | 0.14 |  | Int x freq | 4 | 2.7 | 1.0 | 0.44 |
|  | Residual | 18 | 3.9 |  |  |  | Residual | 18 | 2.8 |  |  |
|  | Transformation: None | C=0.24, p-value=1 | | |  |  | Transformation: None | C=0.30, p-value=0.55 | | |  |
|  |  |  |  |  |  |  |  |  |  |  |  |
| Grazers | Intensity=int | 2 | 2.2 | 0.7 | 0.49 |  | Intensity=int | 2 | 7.1 | 0.9 | 0.44 |
|  | Frequency=freq | 2 | 3.6 | 1.2 | 0.32 |  | Frequency=freq | 2 | 4.5 | 0.5 | 0.59 |
|  | Int x freq | 4 | 7.1 | 2.4 | 0.09 |  | Int x freq | 4 | 7.0 | 0.9 | 0.51 |
|  | Residual | 18 | 2.9 |  |  |  | Residual | 18 | 8.2 |  |  |
|  | Transformation: None | C=0.31, p-value=0.47 | | |  |  | Transformation: None | C=0.42, p-value=0.11 | | |  |
|  |  |  |  |  |  |  |  |  |  |  |  |
| *Rivularia spp.* | Intensity=int | 2 | 0.07 | 2.8 | 0.09 |  | Intensity=int | 2 | 1.7 | 6.4 | **0.01** |
|  | Frequency=freq | 2 | 0.08 | 3.0 | 0.08 |  | Frequency=freq | 2 | 0.2 | 0.6 | 0.58 |
|  | Int x freq | 4 | 0.05 | 1.9 | 0.16 |  | Int x freq | 4 | 0.2 | 0.9 | 0.50 |
|  | Residual | 18 | 0.03 |  |  |  | Residual | 18 | 0.3 |  |  |
|  | Transformation: 1/(x+1) | C=0.43, p-value=0.11 | | |  |  | Transformation: ln(x+1) | C=0.25, p-value=0.91 | | |  |
|  |  |  |  |  |  |  | SNK test: medium intensity>low intensity, medium intensity>high intensity | | | | |
|  |  |  |  |  |  |  |  |  |  |  |  |
| Encrusting algae | Intensity=int | 2 | 24.2 | 0.1 | 0.91 |  | Intensity=int | 2 | 0.03 | 0.8 | 0.46 |
|  | Frequency=freq | 2 | 98.1 | 0.4 | 0.68 |  | Frequency=freq | 2 | 0.05 | 1.5 | 0.25 |
|  | Int x freq | 4 | 564.3 | 2.3 | 0.10 |  | Int x freq | 4 | 0.05 | 1.6 | 0.22 |
|  | Residual | 18 | 245.1 |  |  |  | Residual | 18 | 0.03 |  |  |
|  | Transformation: None | C=0.47, p-value=0.05 | | |  |  | Transformation: 1/(x+1) | C=0.44, p-value=0.09 | | |  |
|  |  |  |  |  |  |  |  |  |  |  |  |
| Filamentous algae | Intensity=int | 2 | 0.572 | 0.6 | 0.54 |  | Intensity=int | 2 | 0.6 | 1.0 | 0.39 |
|  | Frequency=freq | 2 | 0.003 | 0.0 | 1.00 |  | Frequency=freq | 2 | 0.3 | 0.4 | 0.66 |
|  | Int x freq | 4 | 0.445 | 0.5 | 0.74 |  | Int x freq | 4 | 0.1 | 0.2 | 0.92 |
|  | Residual | 18 | 0.895 |  |  |  | Residual | 18 | 0.6 |  |  |
|  | Transformation: ln(x+1) | C=0.29, p-value=0.61 | | |  |  | Transformation: ln(x+1) | C=0.26 p-value=0.82 | | |  |
|  |  |  |  |  |  |  |  |  |  |  |  |
| Complex algae | Intensity=int | 2 | 2.5 | 0.1 | 0.91 |  | Intensity=int | 2 | 13.1 | 0.6 | 0.56 |
|  | Frequency=freq | 2 | 29.4 | 1.1 | 0.34 |  | Frequency=freq | 2 | 11.2 | 0.5 | 0.61 |
|  | Int x freq | 4 | 29.7 | 1.2 | 0.37 |  | Int x freq | 4 | 0.2 | 0.0 | 1.00 |
|  | Residual | 18 | 25.8 |  |  |  | Residual | 18 | 22.1 |  |  |
|  | Transformation: None | C=0.35, p-value=0.3 | | |  |  | Transformation: None | C=0.22, p-value=1 | | |  |
|  |  |  |  |  |  |  |  |  |  |  |  |
| *Chthamalus stellatus* | Intensity=int | 2 | 0.8 | 4.4 | **0.03** |  | Intensity=int | 2 | 1.8 | 5.2 | **0.02** |
|  | Frequency=freq | 2 | 0.4 | 2.1 | 0.15 |  | Frequency=freq | 2 | 0.7 | 2.1 | 0.16 |
|  | Int x freq | 4 | 0.5 | 2.6 | 0.07 |  | Int x freq | 4 | 0.8 | 2.4 | 0.09 |
|  | Residual | 18 | 0.2 |  |  |  | Residual | 18 | 0.3 |  |  |
|  | Transformation: ln(x+1) | C=0.36, p-value=0.25 | | |  |  | Transformation: ln(x+1) | C=0.37, p-value=0.21 | | |  |
|  | SNK test: low intensity>high intensity | | |  |  |  | SNK test: low intensity>high intensity, medium intensity>high intensity | | | | |
|  |  |  |  |  |  |  |  |  |  |  |  |
| *Mytilus galloprovincialis* | Intensity=int | 2 | 2.8 | 0.5 | 0.61 |  | Intensity=int | 2 | 0.03 | 0.4 | 0.67 |
|  | Frequency=freq | 2 | 4.8 | 0.9 | 0.43 |  | Frequency=freq | 2 | 0.03 | 0.5 | 0.64 |
|  | Int x freq | 4 | 3.1 | 0.6 | 0.70 |  | Int x freq | 4 | 0.05 | 0.7 | 0.61 |
|  | Residual | 18 | 5.5 |  |  |  | Residual | 18 | 0.07 |  |  |
|  | Transformation: None | C=0.40, p-value=0.15 | | |  |  | Transformation: 1/(x+1) | C=0.35, p-value=0.30 | | |  |

Appendix 2. Summary of the PERMANOVA, testing the effects of increasing storm intensity on the structure of assemblages of sessile taxa of mid-shore rocky assemblages. Experimental levels comprise a gradient of simulated storms with the same overall intensity, ranging from several small storms to a single large one, and one unmanipulated control. Data was based on Bray-Curtis untransformed dissimilarities. The experiment was run twice, each one beginning at a different time of the year.

| First run |  |  |  |  |  | Second run |  |  |  |  |
| --- | --- | --- | --- | --- | --- | --- | --- | --- | --- | --- |
| Source of variation | df | MS | Pseudo-F | p |  | Source of variation | df | MS | Pseudo-F | p |
| Among experimental levels | 4 | 4239 | 3.14 | 0.001 |  | Among experimental levels | 4 | 1677 | 0.92 | 0.589 |
| Control vs. treatments | 1 | 2031 | 0.92 | 0.483 |  | Control vs. treatments | 1 | 3125 | 1.87 | 0.065 |
| Among treatments | 3 | 4975 | 4.29 | 0.001 |  | Among treatments | 3 | 1194 | 0.73 | 0.77 |
| Residual | 10 | 1348 |  |  |  | Residual | 10 | 1808 |  |  |

Appendix 3. *Post-hoc* comparisons among the treatments that comprises a gradient of simulated storms with the same overall intensity, ranging from several small storms (level 1) to a single large one (level 6) based on the structure of assemblages of sessile taxa of mid-shore rocky assemblages, in the first run of the experiment. For a schematic representation of the experimental design see Appendix 2. Results show the p value calculated using Monte Carlo test. Significant differences among treatments are in bold.

|  | Level 1 | Level 2 | Level 3 |
| --- | --- | --- | --- |
| Level 1 |  |  |  |
| Level 2 | 0.064 |  |  |
| Level 3 | 0.069 | 0.405 |  |
| Level 6 | **0.035** | **0.007** | **0.023** |

Appendix 4. Diagram of the experimental design to test the effects of intensity and frequency of storms through a factorial design (part 1 of the experiment) and along a gradient of simulated storms with the same overall intensity, ranging from several small storms to a single large one (part 2 of the experiment).


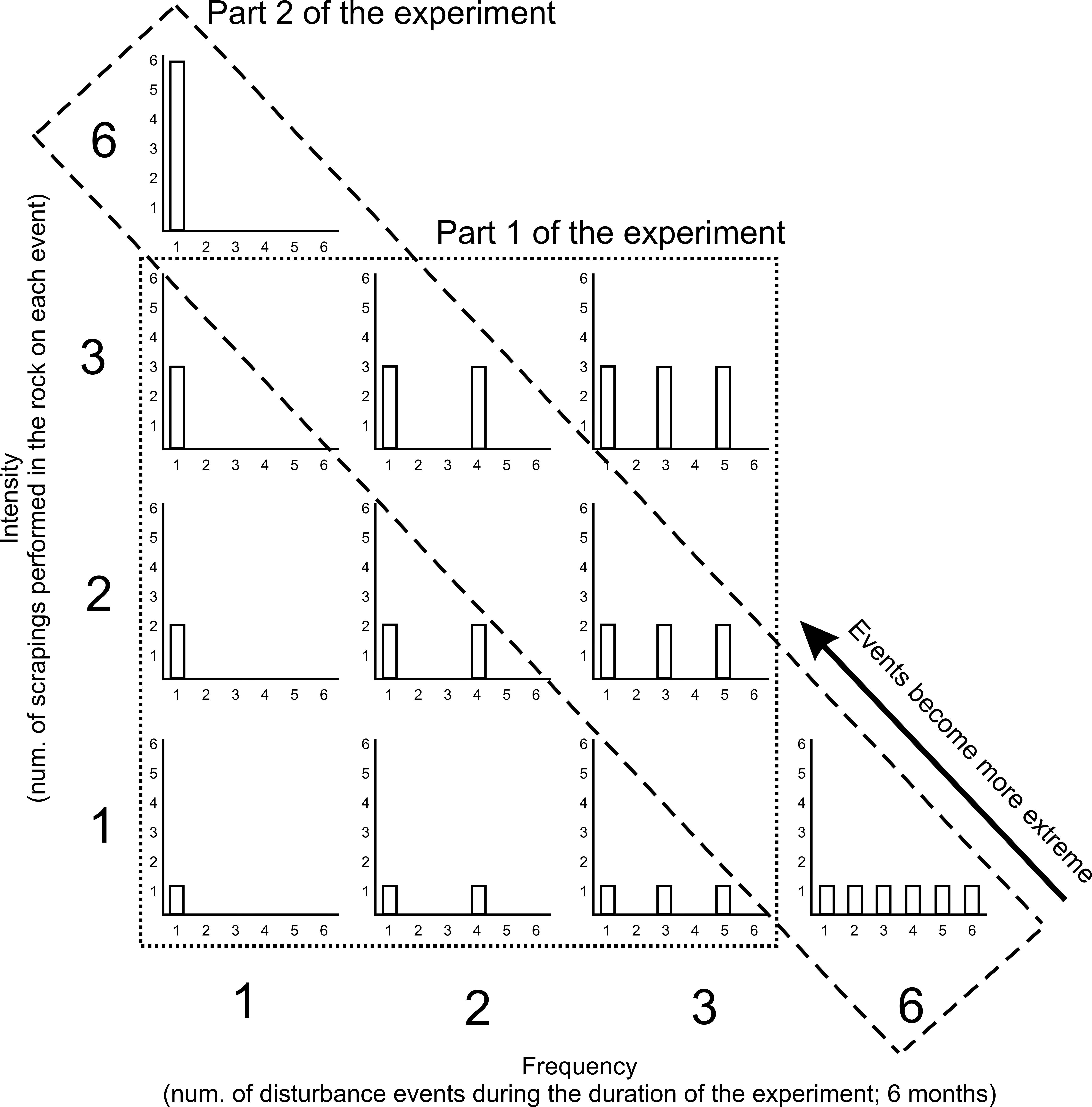


Appendix 5. Temporal profile of the sampling and simulated storm events performed during the two runs of the experiment.


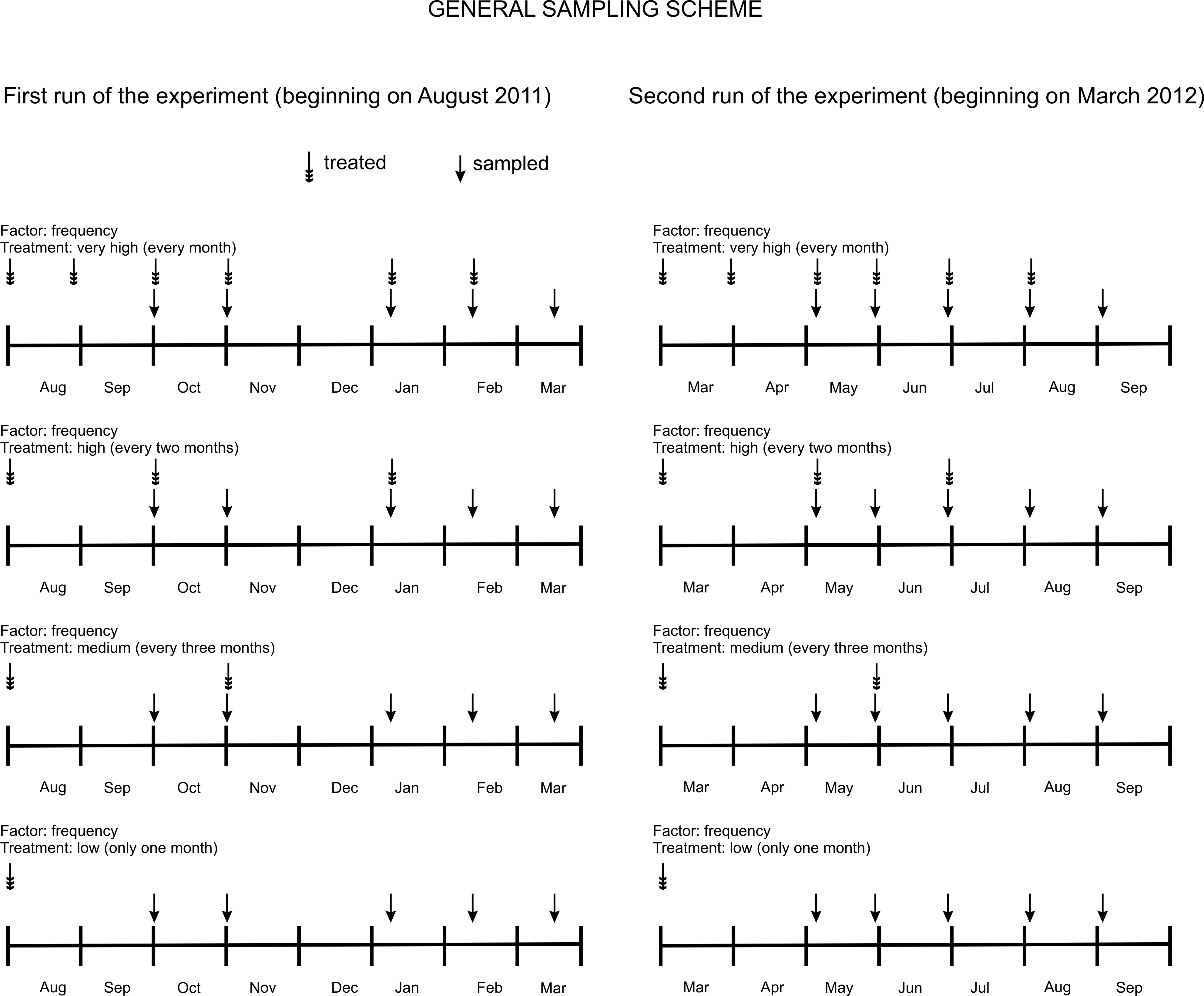

Supplement: Supplementary Information [file srep30607-s1.doc]
